# Supplementary material for: Mapping the evolution of fertility support policies in China: A content and instrumental analysis
Source: PLoS One. 2025 Oct 9;20(10):e0332137. doi: 10.1371/journal.pone.0332137 (PMC12510515; doi:10.1371/journal.pone.0332137)
Supplement: S1 Appendix — (ZIP) [file pone.0332137.s001.zip › S1 Appendix. 226 original policy documents/4-国家人口计生委关于全面加强人口和计划生育科技工作的决定(FBM-CLI-4-78664).doc]

国家人口计生委关于全面加强人口和计划生育科技工作的决定

**发布部门：** [国家人口和计划生育委员会(含国家计划生育委员会)(已撤销)](https://www.pkulaw.com/law/chl?Aggs.IssueDepartment=60304) [机构沿革](javascript:void(0))

**发布日期：**2006.07.24

**实施日期：**2006.07.24

**时效性：** [现行有效](https://www.pkulaw.com/law/chl?Aggs.TimelinessDic=01)

**效力级别：** [部门工作文件](https://www.pkulaw.com/law/chl?Aggs.EffectivenessDic=XE0303)

**法规类别：** [人口计划管理](https://www.pkulaw.com/law/chl?Aggs.Category=09701)

国家人口计生委关于全面加强人口和计划生育科技工作的决定

各省、自治区、直辖市人口计生委，计划单列市、新疆生产建设兵团人口计生委，解放军、武警部队人口和计划生育领导小组办公室，中直机关、中央国家机关人口计生委，委机关各单位，各直属、挂靠单位：
　　
　　为贯彻落实全国科学技术大会精神，全面提升人口和计划生育科学技术工作水平，进一步增强科技创新能力，充分发挥科学技术在新时期人口和计划生育工作中的引领和支撑作用，现作出如下决定。
　　一、充分认识全面加强人口和计划生育科技工作的重要意义
　　30多年的实践证明，人口和计划生育科学技术在落实计划生育基本国策的历史进程中不断发展，在坚持以人为本、促进人的全面发展的工作实践中不断创新，为实现人口再生产类型的历史性转变提供了有力支撑和坚强保障，为人口和计划生育事业持续稳定健康发展作出了突出贡献。
　　当前，我国人口发展和计划生育工作形势不容乐观：稳定低生育水平的任务十分艰巨，出生人口素质亟待提高，出生人口性别比依然偏高，人口老龄化日益严重，劳动年龄人口规模庞大，流动迁移人口持续增加。经过多年的努力，人口和计划生育科技工作取得了长足进展，但依然面临诸多挑战：基层服务网络基础设施、技术装备、人员素质、服务能力与科学发展观的要求不相适应，创新能力、科技成果转化和推广应用能力与人民群众计划生育生殖健康需求不相适应，科技体制机制不完善、改革滞后、投入不足及领军人才缺乏与新时期人口和计划生育工作的新任务不相适应。
　　全面加强人口和计划生育科技工作，为新时期人口和计划生育工作提供引领和支撑，是落实科学发展观，促进人口健康、保障人口安全的重要手段，是落实党中央关于坚持自主创新、建设创新型国家战略部署的具体举措，是稳定低生育水平、统筹解决人口问题、促进人的全面发展的必然要求。必须进一步提高思想认识，增强责任感、使命感和紧迫感，把科学技术放在人口和计划生育工作优先发展的战略地位。

　　二、准确把握全面加强人口和计划生育科技工作的总体要求和基本原则
　　总体要求：
　　坚持以邓小平理论和“三个代表”重要思想为指导，全面落实科学发展观，遵循“自主创新、重点跨越、支撑发展、引领未来”的指导方针，以促进人口健康、保障人口安全为目标，加快构建人口和计划生育科技创新体系，建立和完善人口发展战略研究长效机制、人口和计划生育技术创新机制以及社会管理和公共服务机制，显著提高科技创新能力、综合服务能力以及科技管理能力，全面提升科技工作水平，为稳定低生育水平、统筹解决人口问题、促进人的全面发展提供战略思路和科技支撑。
　　基本原则：
　　--自主创新。把自主创新作为人口和计划生育科技发展的战略重点和事业发展的不竭动力，选择具有一定基础和优势的关键性和战略性重大科技课题，集中攻关。从注重单项技术研发向重大产品的集成创新转变，从以市场换技术向引进、消化、吸收再创新转变，从注重成果的技术指标向注重成果的市场化、产业化转变，在关系经济社会发展的重点领域实现科技创新的新突破。
　　
　　--以人为本。确立人在科技工作中的核心地位，面向全人口，面向社区、家庭，面向生命全过程，以促进人的全面发展和社会均衡发展为宗旨，把科技创新与提高人民生活生命质量紧密结合，与提高人民科学文化素质和健康素质紧密结合，与建设社会主义新农村紧密结合，努力使计划生育家庭和所有社会成员分享科技进步的成果和新的发展机会。
　　
　　--资源共享。根据人口和计划生育科技发展的突出问题和战略需求，按照开放共享、提高效益、保障重点、服务创新的要求，充分发挥人口和计划生育相关科研院所、社会团体和企业单位的优势和特色，打破条块分割、自成体系、相互封闭、重复分散的格局，推进科技资源共享，形成广泛的、多层次的创新合作机制。
　　--分类指导。根据不同地区经济社会发展和计划生育工作水平，因地制宜，发挥比较优势，形成区域特色。科技力量较强的地区和企业，大力开展自主创新，不断创造新的增长点；其他地区着力开展成熟科技成果的转化及适宜技术的引进、推广；从实际出发确定各地区科技工作重点，统筹规划，协调推进。

　　三、着力构建人口和计划生育科技创新体系
　　（一）人口发展战略研究体系。遵循以科学发展观统领人口发展全局的总体思路，科学预测中长期人口发展状况及影响因素，辩证分析人口与经济、社会、资源、环境的相互关系及变动规律，研究提出关系国民经济和社会发展全局的重大决策建议，有效建立具有预期性、导向性的人口与发展指标体系、人口发展综合监测评估体系、人口安全预警预报制度以及信息共享机制，推动国家、区域和省级人口发展战略研究向纵深发展，为稳定低生育水平、统筹解决人口问题、促进人的全面发展和国家宏观决策提供扎实科学的理论依据、战略思路和政策建议。
　　（二）人口和计划生育技术创新体系。以提高群众生活质量和生命质量、强化公共预防保健体系为目标，加强计划生育生殖健康科技自主创新，重点开展计划生育、优生优育、生殖健康基础研究和应用研究，开发自然科学、社会科学和工程科学的集成成果，探索避孕节育新方法，提高避孕药具质量。研发重大出生缺陷干预、生殖道感染干预、不孕不育筛查诊断等适宜新技术，研制更加人性化、个性化的生殖保健新产品，开发生殖健康相关疾病检测诊治的数字化新设备，探索监测胎儿性别鉴定的便捷、安全新方法。加速科研成果转化，加快新知识、新产品、新技术普及、推广和应用。
　　（三）计划生育生殖健康产业发展体系。贯彻“以事业带动产业，以产业促进事业”的战略构想，加强产业发展战略研究。规范计划生育生殖健康产品市场，完善以产品资质认证、质量管理、市场监督为重点的管理制度，加强市场监管。坚持办好计划生育生殖健康新技术新产品博览会。建立和充分发挥生殖健康产业协会的作用。建立适应市场经济体制、促进企业自主创新的政府采购制度，发挥政府采购的导向作用，扶持和推动生殖健康产业基地建设。利用人口和计划生育工作网络优势，推动生殖健康产业的拓展提升和产品的更新换代，不断满足人民群众计划生育、优生优育、生殖健康和家庭和谐的需求。
　　（四）人口和计划生育管理与服务质量监控体系。健全以《[计划生育技术服务管理条例](https://www.pkulaw.com/chl/b5b4877bc6cd96cabdfb.html)》、《计划生育手术常规》和《计划生育技术服务质量管理规范》等为主体的法规规章和标准规范，严格执行计划生育技术服务机构、人员准入制度以及执业资格制度，完善技术服务机构质量管理制度，开展计划生育技术服务质量评估，强化执法监督。研究并制定适用于我国人群的生殖健康评价标准，建立计划生育生殖健康新技术新产品准入制度、风险效益评估和报告制度，加强避孕药具不良反应监测和长期应用安全性研究，推动建立计划生育生殖健康产品技术标准，完善以产品资质认定、质量管理、市场监督为重点的管理制度。

　　四、加快建立和完善人口和计划生育科技创新长效机制
　　（一）人口发展战略研究机制。不断完善“政府导向、部门合作、专家为主、课题运作”的人口发展战略研究长效机制。健全人口发展战略研究调控机制，科学设计重点课题、研究方案，形成科学权威的研究成果；建立人口发展战略研究协调机制，充分发挥政府部门组织、协调和服务职能，建立统筹国家、区域、省级人口发展战略研究和部门间沟通合作制度，开展联合攻关，实现资源共享；建立人口发展战略研究专家库，加强学术交流，以专家为主体，开展课题研究，进行成果评审；培育人口发展战略研究基地，发挥各类科研机构和高等院校的优势，注重培养年轻学者，拓展研究视野，造就具有战略思维、世界眼光和科学民主态度的研究队伍和管理队伍。
　　（二）技术创新和产品开发机制。建立“政府引领、着眼需求、企业为主、联合攻关”的技术创新和产品开发长效机制。政府通过制定科技发展战略和规划、组织重大科技活动，构建创新平台；深化人口和计划生育科研管理体制改革，建立政府宏观调控与市场配置资源相结合、制度创新与政策配套相结合、推进系统整合与激发微观活力相结合的机制；鼓励企业加快研究开发机构建设和加大研究开发投入，承担研究开发任务，参与重大科技攻关。加强科技信息资源平台和科技成果产权交易平台建设，建立科技成果信息共享机制，完善技术转移机制，加快企业对科研成果的应用转化和产业化，促进以市场为导向、以企业为主体，产学研一体化的自主创新体系的形成。进一步优化重点实验室、研究中心布局，建设具有国际先进水平的计划生育生殖健康科研基地，组建若干多学科交叉的国家重点实验室和工程技术研究中心，以科技创新为先导，以服务网络为载体，形成事业和产业相互促进的局面。
　　（三）社会管理和公共服务机制。建立“目标一致、主体多元、公开透明、便捷高效”的人口和计划生育社会管理和公共服务长效机制。政府部门要强化社会管理职能和公共服务意识，创造良好制度环境，提供关键性、普惠性的公共服务，实施人口和计划生育社会管理和公共服务全程监测。明确计划生育技术服务机构是公共服务和公共卫生体系的重要组成部分，是公益性全额拨款事业单位，要坚持计划生育行业特色不改变，坚持稳定机构和队伍不动摇。将计划生育技术服务网络建设纳入各地经济社会发展规划中，协调相关部门，制定有利于服务网络发展的政策和措施。坚持“面向基层、深入乡村、服务上门、方便群众”的工作方针，强化宣传教育、技术服务、信息咨询、人员培训、药具发放五大功能，切实加强计划生育服务网络标准化、规范化建设和队伍职业化建设；推动基层组织民主自治，调动社会成员直接参与公共事务的积极性，将自上而下的行政管理模式和自下而上的监督模式相结合，畅通政府与社会沟通和民意表达的渠道；充分发挥计划生育协会等社会团体的作用，更好地推进社会管理和公共服务。

　　五、切实加强对人口和计划生育科技工作的组织领导
　　（一）加强组织领导。将科技工作纳入各级人口和计划生育工作领导小组的重要议事日程，定期听取科技工作汇报，适时召开会议专题研究，积极创造有利于人口和计划生育科技工作发展的政策环境，科学制定完善本地人口和计划生育科技工作规划，主要领导“亲自抓、负总责”，切实解决科技工作的重要问题。把推动科技创新纳入人口和计划生育目标管理责任制，加大监督检查和考核力度。各级人口和计划生育部门要成立科技工作领导小组，加强与同级政府科技主管部门的业务联系。人口和计划生育部门分管科技工作的领导要具有医学专业背景，科技管理部门要配备懂医学、会管理、综合素质高的人员，计划生育技术服务机构的法人代表要具有医学专业学历。
　　（二）加强人才队伍建设。建立数量充足、结构合理、学科健全的高素质的科技人才队伍。引进、培养、用好学术带头人和学科领军人物，努力营造人尽其才、才尽其用的体制环境。改进科技人才的评价体系，建立科学的选人用人机制，完善对科技人员的激励机制，加大对做出突出贡献的杰出科技人才的奖励。加快调整技术服务机构人员结构，加强技术服务队伍职业化建设。实施“三千人才”工程，带动和加强技术人员专项培训，建立以专业教育为基础，以岗位培训、继续教育为重点的职业化教育体系。
　　（三）加大经费投入。建立以政府投入为主体的财政投入体系。把公益性科研院所和计划生育技术服务机构的经费纳入财政预算，并不断增加投入；建立健全监督考评机制，确保计划生育免费技术服务经费的落实。落实国债资金项目，支持农村计划生育服务站基础设施建设；争取国家更多重大专项资助，在服务站建设、人才培养、政策支持等方面向中西部倾斜。各级人口和计划生育部门要调整经费支出结构，设立专项，重点向科技工作倾斜，在各级人口和计划生育事业经费中设立服务站建设专项经费和生殖健康促进计划专项经费。围绕国家科技规划纲要，积极争取国家重大科研项目，以项目带动投入。鼓励科研单位与企业合作，引导企业成为科技创新的投入主体。探索从国内外非政府组织、基金会和民间募集科技发展基金的途径，争取建立资金来源多元化的国家人口和计划生育科技发展基金。
　　（四）加强国际交流合作。积极扩大国际和地区科技交流与合作，充分利用全球科技资源，积极引进国际项目、先进技术和管理理念，鼓励和支持科研机构、科技人员参与人口和计划生育生殖健康领域的国际学术交流与合作。鼓励有实力的科研院所与国外研究机构建立联合实验室或研发中心，实施国际合作项目。把人口和计划生育科技合作纳入人口与发展南南合作框架体系，重点着眼于发展中国家的市场和需求，通过多种途径，将我国具有自主知识产权的技术和产品推向国际市场，通过交流与合作，促进科技创新和共同发展。
　　全面加强人口和计划生育科技工作，任务光荣而艰巨。各级人口和计划生育部门要认真贯彻本决定，结合本省（区、市）实际，制定有关配套措施，协调解决执行过程中出现的问题，推动人口和计划生育科技工作全面进步，推动人口和计划生育事业持续稳定健康发展。

　　二00六年七月二十四日

© 北大法宝：（[www.pkulaw.com](https://www.pkulaw.com/)) 专业提供法律信息、法学知识和法律软件领域各类解决方案。北大法宝为您提供丰富的参考资料，正式引用法规条文时请与标准文本核对。
欢迎查看所有[产品和服务](https://www.pkulaw.com/helps/2.html)。法宝快讯：[如何快速找到您需要的检索结果？](https://www.pkulaw.com/helps/70.html)[法宝](https://www.pkulaw.com/helps/69.html)V6有何新特色？

扫描二维码阅读原文

**原文链接：**<https://www.pkulaw.com/chl/07f4ea04cb0d57b1bdfb.html>
